# Effect of Rare Earth Elements on Microstructure and Tensile Behavior of Nb-Containing Microalloyed Steels

**DOI:** 10.3390/ma17071701

**Published:** 2024-04-08

**Authors:** Shi Cheng, Tingping Hou, Yihang Zheng, Chaochao Yin, Kaiming Wu

**Affiliations:** The State Key Laboratory of Refractories and Metallurgy, Hubei Province Key Laboratory of Systems Science on Metallurgical Processing, International Research Institute for Steel Technology, Collaborative Center on Advanced Steels, Wuhan University of Science and Technology, Wuhan 430081, China; 202102601043@wust.edu.cn (S.C.); zhengyihang@wust.edu.cn (Y.Z.); ycc0125@126.com (C.Y.)

**Keywords:** rare earth element, low-carbon steel, tensile properties, microstructure

## Abstract

The present investigation endeavors to explore the influence of rare earth elements on the strength and plasticity characteristics of low-carbon microalloyed steel under tensile loading conditions. The findings from the conducted tensile tests indicate that the incorporation of rare earths leads to a notable enhancement in the yield strength, ultimate tensile strength, and ductility properties of the steel. A comparative analysis of the microstructures reveals that the presence of rare earths significantly refines and optimizes the microstructure of the microalloyed steel. This optimization is manifested through a reduction in grain size, diminution of inclusion sizes, and a concomitant rise in their number density. Moreover, the addition of rare earths is observed to foster an increase in the volumetric fraction of carbides within the steel matrix. These multifaceted microstructural alterations collectively contribute to a substantial strengthening of the microalloyed steel. Furthermore, it is elucidated that the synergistic interaction between rare earth elements and both carbon (C) and niobium (Nb) in the steel matrix augments the extent of the Lüders strain region during the tensile deformation of specimens. This phenomenon is accompanied by the effective modification of inclusions by the rare earths, which serves to mitigate stress concentrations at the interfaces between the inclusions and the surrounding matrix. This article systematically evaluates the modification mechanism of rare earth microalloying, which provides a basis for broadening the application of rare earth microalloying in microalloyed steel.

## 1. Introduction

High-strength microalloyed steel (HSLA) is widely utilized as the main material for offshore platforms, offshore wind power generation, and other marine engineering equipment based on its high strength, high toughness, and excellent fatigue properties [1,2,3]. Conventional HSLA steels are mainly alloyed with alloying elements such as Nb, Ti, and Cr to improve the strength and plasticity of the materials. The formation of diffusely distributed nanosized MC carbides through microalloying can contribute to grain refinement, inhibition of dynamic recrystallization, and strengthening of the matrix [4,5,6]. The addition of strong carbide-forming elements (Nb, Ti, Mo, etc.) affects the diffusion and distribution of carbon (C) atoms, which in turn affects carbide formation. In order to further improve the strength of microalloyed steels, modern industry has improved the material properties by adding rare earth elements to modify the inclusions in the steel [7,8]. The oxygen and sulfur content of steel can be successfully reduced by rare earth (RE) metals due to their great affinity for these components [9]. Reducing the inclusions and the stress concentration between the matrix is made possible by the control of oxygen sulfide in RE elements, which results in small-sized spherical RE-O-S or RE-S inclusions [10,11]. The impact of inclusions modified with rare earth elements on the tensile characteristics of steel has been the subject of numerous investigations [12,13,14].

In addition, small amounts of rare earth elements are dissolved in microalloyed steels by vacancy diffusion, occupying lattice cross-section points. In manganese steels, rare earth elements interact with carbon atoms and influence the phase transformation and precipitation of carbides [14,15,16]. Rare earth atoms with large diameters and high distortion energies tend to polarize at the ferrite–carbide interface [16,17]. The microalloying effect of rare earths in steel is becoming increasingly significant as the cleanliness of steel continues to improve. By adding rare earths to AISI D2 steels, M_7_C_3_ carbides were refined and uniformly distributed, resulting in a 75% increase in impact toughness [18,19]. Although rare earths have been extensively studied in the field of microalloyed steels, there have been fewer studies devoted to explaining the mechanism for the effect of rare earths on plasticity. Moreover, previous studies have mainly focused on explaining the increase in yield strength by rare earths and have shown that rare earths have less of an effect on tensile strength [20,21,22].

Rare earth microalloyed steel is essential for the improvement of material properties. This study focuses on comparing the effects of rare earth additions on material properties. The microstructures of steels with and without rare earths were systematically studied with characterization tools such as scanning electron microscopy and electron microscopy. Microstructural analysis provides a complete explanation of the increase in the strong plasticity (yield strength, tensile strength, and plasticity) as well as the tensile behavior (the Lüders effect/yield-point phenomenon) exhibited by the materials during the tensile process.

## 2. Experimental Details

### 2.1. Experimental Materials and Processes

The steels designed for the experiment were 0.05C and RE-0.05C low-carbon steels, and their chemical compositions are given in Table 1. The research steels were prepared using normal metallurgical processes, including iron pre-treatment, basic oxygen furnace (BOF) treatment, ladle arc refining furnace (LF) treatment, RH vacuum degassing, and continuous casting processes. To minimize the different impurity elements in the raw materials, the pure iron operation was selected to manufacture high-quality low-carbon steels. Initially, the hot-rolled steel plates were alloyed with ferrosilicon after being deoxidized, mostly to prevent the addition of carbon. The basic chemical composition of the hot-rolled steel plates was measured by the optical emission spectroscopy (OES; ARL 3460, Kronach Zahner, Germany) technique. When rare earth elements are added to steel in a vacuum atmosphere, the oxygen content of the steel is reduced to a low level (20–40 ppm) using a rapid oxygen determination probe. To assure uniformity of the material, rare earths were added to the steel using secondary refining and an argon blowing process at the bottom of the ladle. The final thickness of the steel sheet material was 10 mm. Inductively coupled plasma (ICP-MS; ICAPQ, Kronach Zahner, Germany) was used to measure the amounts of rare earths still present in the castings [23,24]; the results are shown in Table 1.

### 2.2. Microstructural Characterization

The steel samples were prepared according to standard metallographic procedures prior to microstructural examination. The scanning electron microscopy samples were prepared by grinding at different magnifications and polishing with diamond gesso. Specimens for microstructural characterization were etched using 4% nitric acid alcohol, and EBSD samples were electrolyzed on the sample surface by means of a 5% ethanol perchlorate solution. In order to observe the three-dimensional morphology of inclusions, the specimen preparation for the observation of inclusions in steel was performed by electrolyzing a 10 × 10 × 2 mm^3^ sample in a non-aqueous solution. The specimens were electrolyzed for 10 min in a non-aqueous solution (pH ≈ 8) from 0 to 5 °C. A portion of the matrix was electrolyzed to expose the inclusions. After electrolysis, the specimens were passed through alcohol several times to remove the residual electrolyte from the surface of the specimens. The inclusions of the samples under polishing conditions were observed with a scanning electron microscope (SEM; QUANTA 450, Lincoln, NE, USA) in secondary electron (SE) and backscattered electron (BSE) modes to obtain a better contrast of the inclusions. The microstructure and niobium-containing second-phase precipitates were examined using an HRTEM JEM-2100 (Akishima, Japan) equipped with an Energy Dispersive Spectrometer (EDS) (test voltage: 200 kV). The samples used were made of 3 mm diameter discs that had been ground to a thickness of about 80 µm. The small discs were electrolytically polished at room temperature and at a voltage of 40 V. Dislocation density was quantified by X-ray diffraction (XRD) using a Cu-Kα radiation source (λ = 1.5405 Å). The scan range was between 40° and 100°, with a step size of 0.02°.

### 2.3. Tensile Tests

The mechanical properties of the tensile samples were tested using an MTS E45.305 (Meters Industrial Systems, Shenzhen, China) tensile testing machine with a strain rate of 2 mm/min at room temperature. Three tensile tests were performed on each sample to ensure the accuracy of the tests. Small tensile specimens were prepared in accordance with ASTM E8-04 [25] and were carefully ground prior to testing to remove any scratches remaining from the specimen preparation process. During the tensile tests, load–displacement curves were continuously recorded and converted to engineering stress–strain curves. The recorded displacements corresponded to the displacements determined by the movement of the machine beam.

## 3. Results

### 3.1. Microstructure Characteristics of RE-Containing and RE-Free Steels

**Figure 1:**
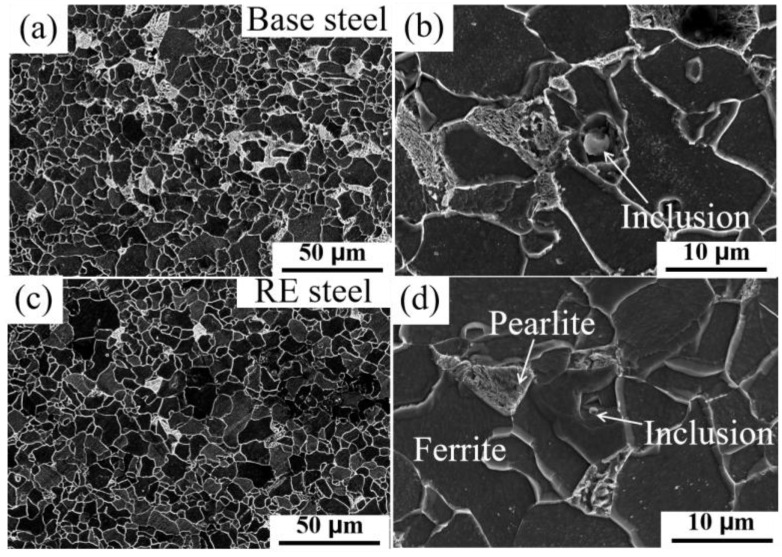
Micrographs of basic steels (**a**,**b**) and rare earth microalloyed steels (**c**,**d**).

**Figure 2:**
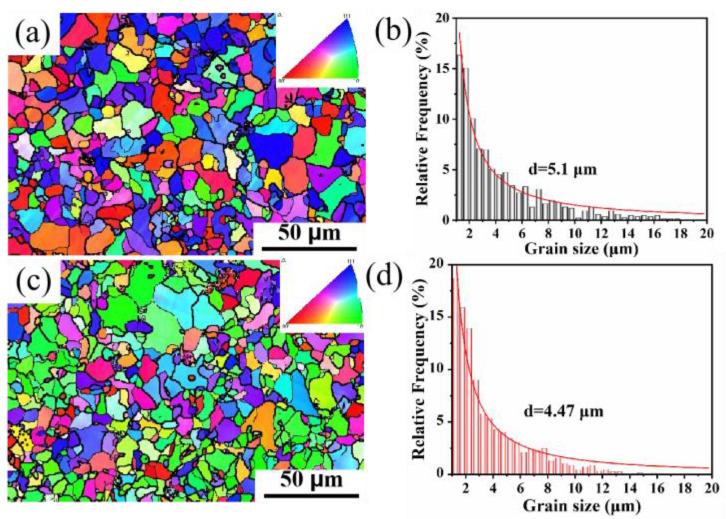
Inverse pole diagrams and grain size distributions of base steel and rare earth microalloyed steel: (**a**,**b**) base steel microstructure; (**c**,**d**) rare earth microalloyed steel microstructure.

The microstructure of microalloyed steel directly affects the mechanical properties of the steel, and the refinement of ferrite grains and pearlite is beneficial with respect to the tensile and toughness properties [26,27]. Figure 1 shows the microstructure consisting of a two-phase organization of ferrite + pearlite. A finer distribution of pearlite can be observed in the microstructure of the rare earth microalloyed steel (Figure 1c) compared to the base steel (Figure 1a). Based on the statistical data from SEM images, the area fraction of pearlite in the base steel is about 21.6%, while that in the rare earth microalloyed steel is about 21.1%. The characterization data of the microscopic volume fraction for microstructure statistics are presented in Table 2. It indicates that the addition of rare earths can effectively optimize the ferrite and pearlite. It has been observed that the mechanism of refinement by rare earths is primarily explained by the fact that rare earth inclusions act as heterogeneous nucleation sites during solidification, which can effectively refine the austenite grain size (PAGS) [28,29]. In order to verify the organization of the materials even further, the EBSD data with and without rare earth materials were compared (Figure 2). Figure 2a,c show a small-change microstructure distribution with the addition of rare earths. According to the EBSD grain-size statistics (Figure 2b,d), the average grain sizes of the base steel and the rare earth microalloyed steel are about 5.01 µm and 4.47 µm, respectively, which indicates the refinement behavior in RE microalloyed steel; this is similar to other reports in the literature [30].

In addition, the shape, size, and distribution of inclusions in microalloyed steels have a significant effect on the material properties [31,32]. Figure 1b,d illustrate the presence of inclusions with sizes around 3 μm in two microalloyed steels. Since the organization was obtained by corrosion with acidic solution, the full shape of the inclusions obtained could not be observed. To further investigate the effect of rare earth microalloying on the organization, the inclusions were partially exposed by electrolysis of the substrate with non-aqueous solution, and the EDS technique was used to characterize the inclusions in the microalloyed steels, which will be discussed in the next section.

### 3.2. Characteristics of Inclusions

**Figure 3:**
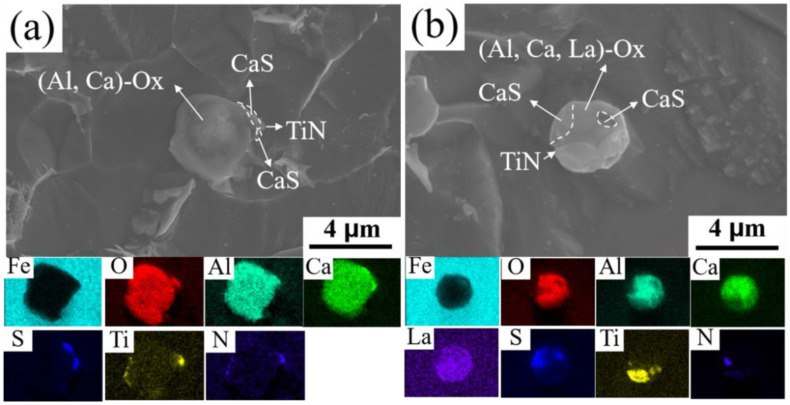
Inclusion morphology and EDS plots: (**a**) base steel; (**b**) rare earth microalloyed steel.

**Figure 4:**
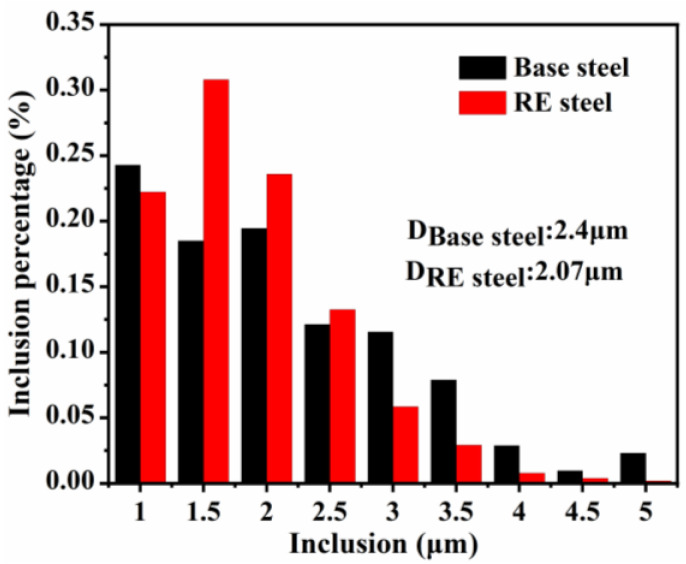
Statistical particle size distribution of inclusions in microalloyed steel.

Inclusions have a significant impact on the performance of microalloyed steel, and rare earth elements can effectively modify inclusions [14,33]. In order to study the effect of rare earths on inclusions, by comparing Figure 3a,b, it can be found that the inclusions in the steel gradually tend to become spherical after the addition of rare earths. Analyzing the composition of inclusions in the microalloyed steel against EDS shows that the inclusions in the base steel consist of (Al, Ca)O oxides as the core, with CaS and TiN attached to the oxides to form the nucleus. The addition of rare earths changes the composition of inclusions. Features such as the number density and particle size of inclusions in microalloyed steels were statistically characterized using the statistical program for inclusions in scanning electron microscopy [30]. The statistical results, including the number densities and particle sizes of inclusions in the basic and rare earth microalloyed steels, obtained by means of SEM are shown in Figure 4. The overall area during SEM scanning was 27.46 mm^2^, and the inclusions’ particle size statistics were above 1 μm. Figure 4 reveals that the inclusions in both microalloyed steels are mainly in the range of 1–2.5 μm. The inclusions in the rare earth microalloyed steel have the smaller particle size of 2.07 μm, while the inclusions in the base steel have a particle size of 2.4 μm. In addition, the rare earth microalloyed steel has a higher density of inclusions (13.5/mm^2^) compared to 10.5/mm^2^ the base steel. This indicates that the inclusions in the rare earth microalloyed steel are finer and more densely distributed, such that they can effectively pin down the austenite grain boundaries and refine the organization [34].

### 3.3. Calculation of Dislocation Density

The interaction of nanocarbides with dislocations is crucial for the strengthening of microalloyed steels. To investigate the effect of rare earths on the strengthening of microalloyed steels, the dislocation densities of basic and rare earth microalloyed steels were analyzed. The dislocation density of ferrite grains was measured by X-ray diffraction (XRD) according to the method originally developed by Williamson and Hall [35,36]. Assuming that the strain within the grain is generated by dislocations only, the dislocation density (ρ) was evaluated by the modified Williamson–Hall (MWH) method [35,36]:

(1) $$∆K=\frac{0.9}{L}+\sqrt{0.5\pi A^{2}b^{2}}\sqrt{\rho }\left(K\sqrt{\bar{C}}\right)$$

where K = 2sin θ/λ is a reciprocal of the lattice spacing, ΔK = 2cos θ(Δθ)/λ is the half-height width of the corresponding peaks plotted according to K, θ is the Bragg diffraction angle, b is the Burgers vector, Δθ is the half-height width of the peaks plotted according to θ, and λ is the wavelength of the X-rays. L is the average size of coherent scattering domains, which can be considered to be the average size of the sub-structure of a dislocation. A is a constant determined by the outer cutoff radius of the dislocation [30].

**Figure 5:**
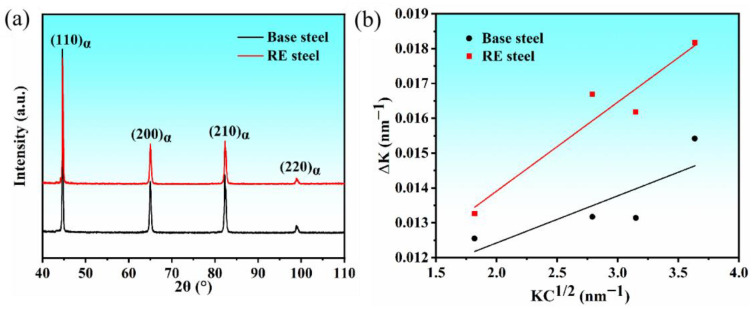
(**a**) Measured X-ray diffraction patterns. (**b**) Williamson–Hall plots of the diffraction patterns for the test steel.

The representative XRD profile (Figure 5a) reveals that the characteristic peaks are mainly shown as four bcc α-Fe peaks ({110}, {200}, {211}, and {220}) of the matrix organization. Detailed XRD peak profiling was utilized to determine the dislocation density in Figure 5b according to the modified Williamson–Hall (MWH) method (Equation (2)) [35,37]. The results indicate that the dislocation density is 3.15 × 10^14^ m^−2^ in the base steel and 3.31 × 10^14^ m^−2^ in the rare earth microalloyed steel, suggesting that the addition of rare earths has an effect on the dislocation density in the steel.

### 3.4. Nanoprecipitation Characterization

**Figure 6:**
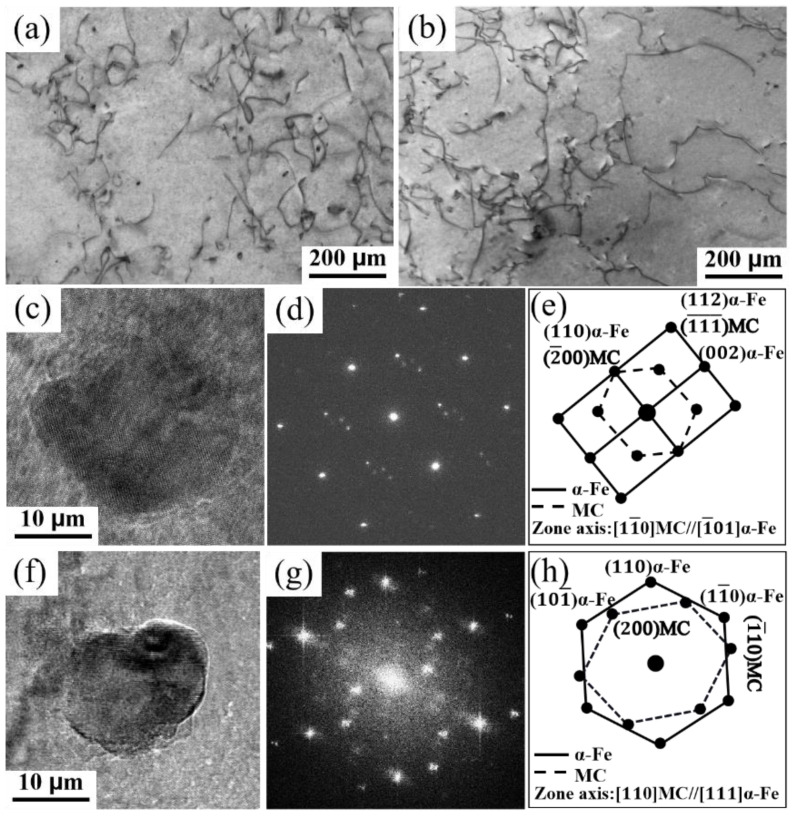
Micromorphology and carbide selection diffractograms of microalloyed steels: (**a**,**c**–**e**) base steel; (**b**,**f**–**h**) rare earth microalloyed steel.

The microstructural characterization of the matrix steel (Figure 6a,c–e) and rare earth microalloyed steel (Figure 6b,f–h) was exhibited by transmission electron microscopy. Both Figure 6a,b show the interaction of nanoprecipitates with dislocations in the steel, which plays a vital role in strengthening the steel. The study of nanocarbides revealed that both steel carbides are MC carbides. Figure 6c–e show the morphology and crystal diffractograms of the nanocarbides in the base steel and the selected diffraction patterns (Figure 6d,e). The nanocarbides exhibit an axial relationship with the matrix as [1$\bar{1}$0]MC//[$\bar{1}$01]α-Fe. Figure 6f,h show the morphology and crystal diffractograms of nanocarbides in the rare earth microalloyed steels, which demonstrate that the nanocarbides exhibit an axial relationship with the matrix as [110]MC//[111]α-Fe. The statistical volume fraction of the precipitates in the rare earth microalloyed steel was obtained as 0.17% by counting several sets of TEM images. However, the volume fraction of MC precipitates in the matrix steel was only 0.11%, and the size of the precipitates increased slightly (Figure 6c,f). This result demonstrates that RE promotes the precipitation of nanocarbides within the grains, which effectively hinders dislocation movement. Chun et al. [38] suggested that nanoscale precipitation can strengthen the matrix in two ways, i.e., preventing dislocation migration through the pinning effect and strengthening through dispersion.

### 3.5. Effect of RE on Tensile Properties of Microalloyed Steel

**Figure 7:**
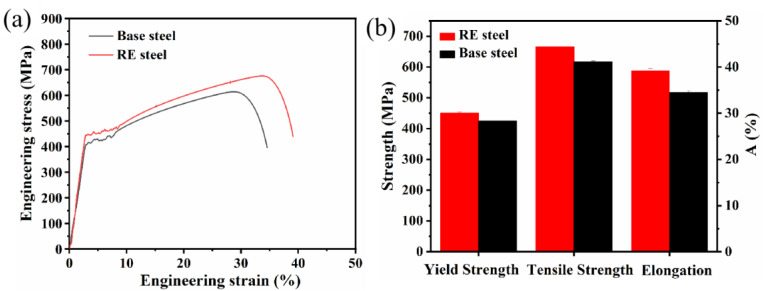
(**a**) Engineering stress–strain curves of microalloyed steel. (**b**) Strength and ductility diagram of microalloyed steel.

Figure 7a presents the engineering stress–strain curves of the matrix steel and the rare earth microalloyed steel at room temperature. From Figure 7a, it can be seen that the tensile curves of both steels start from the elastic region and then reach the yield point. After these different upper and lower yield points, the tensile curves show complex rheological behavior. The shape of this region (the Lüders region [36]) varies depending on the steel under study. The phenomenon of the yield point in tensile testing is often referred to as the Lüders effect, through which the plastic deformation zone is regarded as the Lüders zone [39]. After traversing the Lüders band, the curve persists in its typical behavior, the stress level progressively increasing until it attains the pinnacle referred to as the ultimate tensile strength (UTS). After that, the stress level decreases during the necking process until it finally fractures.

Figure 7b indicates that the yield strength, tensile strength, and elongation of the matrix steel were 425.1 ± 6.2 MPa, 610.4 ± 11.3 MPa, and 34.6 ± 0.8%, respectively. In contrast, the yield strength, tensile strength, and elongation of the rare earth microalloyed steel reached 450 ± 6.0 MPa, 679.6 ± 5.9 Mpa, and 39.1 ± 1.0%, respectively, indicating that the addition of rare earth elements increased the yield strength, tensile strength, and elongation of the steel [36].

## 4. Discussion

**Figure 8:**
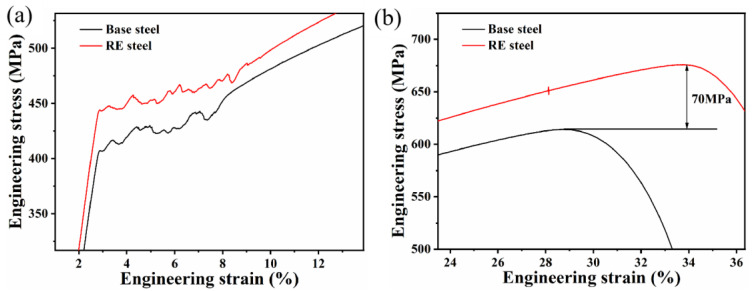
Local enlarged diagram of engineering stress–strain curves: (**a**) Lüders zones; (**b**) tensile strength points.

Figure 7 displays the mechanical properties of the two microalloyed steels, indicating that the addition of rare earths increased the total elongation of the steels and increased the yield and tensile strengths. In addition, both steels exhibited discontinuous yielding and the Lüders effect, as shown in Figure 8a. In line with earlier research, it has been observed that rare earth microalloyed steels exhibit a broader Lüders zone. It was demonstrated that the Lüders strain tends to increase as the ferrite grain size decreases at a specific temperature [40,41]. Furthermore, Varin et al. [42] demonstrated that the presence and distribution of fine precipitates widens the Lüders zone. Both results can explain the wider region of Lüders deformation obtained for rare earth microalloyed steel steels. In addition, the yield strength of the rare earth microalloyed steel was about 50 MPa higher than that of the base steel.

It is well known that the yield strength of a material is influenced by the solid-solution element content, the size of the phases, and the dislocation density, as well as the carbide size and density. Based on the previously described differences between the two microalloyed steels, the main factor responsible for the increase in the strength of rare earth microalloyed steels is grain refinement with an enhancement of carbide and dislocation interactions [43,44,45]. Similarly, it has been noted that the addition of rare earths increases the precipitation of NbC nanoprecipitates and contributes to the precipitation of carbides in microalloyed steels [46]. Pinning of dislocations by niobium nanoprecipitates has been reported to increase the stress in the Lüders zone, contributing to the formation of Lüders bands at higher stress levels and leading to higher yield points [47,48].

In addition, rare earths have an important effect on the phase transition point [49], while the addition of rare earth elements narrows the range of the pearlitic transition, which makes it easy to form finer pearlites (Figure 1). The cleaning of grain boundaries by rare earths increases the strength of grain boundaries and changes the fracture mode of a material [50]. All these factors favor the increase in the strength of the material. Thus, the increase in the yield strength of steel with added rare earths is due to the synergistic effect of multifaceted factors.

Figure 8b unveils a significantly more pronounced enhancement in the UTS of the rare earth microalloyed steel, depicting a notable increase of 70 MPa. To evaluate the difference in tensile strength between the two steels, the work-hardening rates of the two steels were calculated using Equation (2) [51].

**Figure 9:**
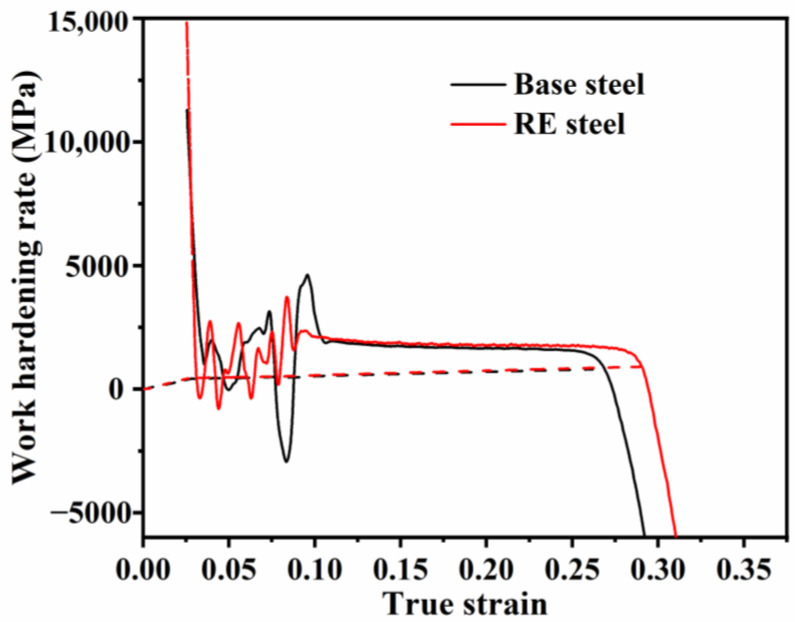
True-strain–work-hardening curve.

(2) θ=dσ/dε

where σ and ε are the true stress and the true strain, respectively. Figure 9 presents the work-hardening rate versus the true-strain curves for the two groups of specimen steels. The work-hardening rate curves are divided into three main parts. The hardening rate of both steels decreases rapidly in the first stage, which is related to the tendency of dislocations to slip in a single system. The decrease in the work-hardening rate is greater in the RE-added steel compared to the matrix steel. This is mainly due to the difference in the precipitate content between the two steels, with the volume fraction of precipitates in the RE-added steel being higher than that in the matrix steel. When dislocations break the blockage of the hard MC carbides and slide (i.e., yield) in the soft ferrite, the stress decreases. As a result, the decrease in the work-hardening rate is greater in RE-added steels.

In the second stage (S2), the work-hardening rate fluctuates considerably for both steels due to the Lüders strain effect, with the difference that it fluctuates even more for the matrix steel due to the uneven distribution of nanoprecipitates (Figure 6) and interstitial atoms (e.g., C atoms) in the matrix steel.

In the third stage (S3) of the work-hardening curve, dislocations and precipitated particles induce a pinning effect, which causes dislocations to continuously slide from the intracrystalline space to the grain boundaries, resulting in dislocations aggregating and entangling at the grain boundaries [52]. However, rare earth atoms also interact with carbon atoms and dislocations, and free electrons move from the lattice compression region to the stretching region, forming localized electric dipoles [8]. Unlike the valence electrons of iron atoms, these free electrons form positive ions that interact with dislocations via short-range electrostatic forces [12]. In addition, interactions between REs, carbon atoms, and dislocations lead to localized aggregation in the extended dislocation-forming layer, hindering dislocation migration [15]. As a result, the work-hardening index of the rare earth microalloyed steel is higher than that of the base steel, which increases the tensile strength of the rare earth microalloyed steel [53].

Figure 7b demonstrates that the addition of rare earths can increase the total elongation of microalloyed steel from 34.6% to 39.1%, where grain size has a significant effect on the elongation of microalloyed steel. It has been suggested that the uniform elongation decreases with decreasing grain size [54,55]. However, according to a previous study [54], the grain size of microalloyed steel decreased after the addition of rare earths. Therefore, besides considering the effect of grain size on the elongation of microalloyed steels, it has been suggested that the morphology and size of inclusions in microalloyed steels also affect the elongation of steel [56]. The significant stress concentration occurring between irregular inclusions and the matrix renders the material susceptible to premature failure under external stresses, consequently diminishing its elongation. Spherical inclusions that keep good contact with the matrix favor steel elongation.

**Figure 10:**
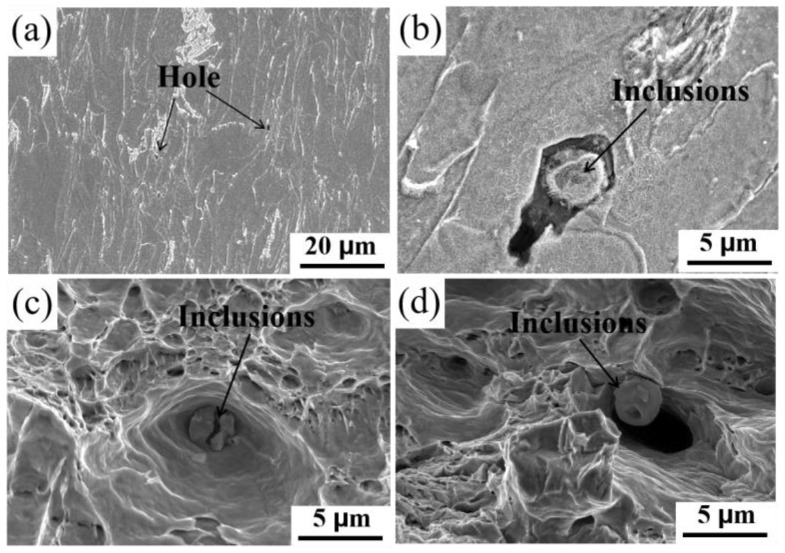
The fractographical microstructure after stretching: (**a**,**b**) cross-section morphology of the basic steel; (**c**,**d**) fracture morphology of the basic steel and the rare earth steel, respectively.

Figure 10a,b show the microscopic morphology of the base steel after stretching, where the deformation behavior of ferrite and pearlite provides a favorable deformation mechanism for higher plasticity. Figure 10b shows the crack initiation between inclusions and the base after stretching, which leads to the early fracture behavior of the material. Figure 10c,d show the port microstructure of the base and rare earth steels after tensile fracture, respectively. Both steels show deep dimples, demonstrating the good plasticity of the material.

However, the broken inclusions deep in the dimples of the base steel indicate that there is a large stress concentration between the inclusions and the matrix, which reduces the plasticity of the material. Rare earth inclusions have better plasticity [9], and there is a gap between the dimples and the inclusions, which provides plastic space in the tensile process. The modification of inclusions by rare earth elements effectively reduces the size of the inclusions and reduces the stress concentration between the inclusions and the matrix, which helps to increase the plasticity of the material. In addition, the Cottrell atmosphere formed by the aggregation of carbon atoms facilitates the expansion of the Lüders strain region to increase the ductile shape of the material [28].

## 5. Conclusions

In this study, the influence of rare earth elements on the properties of microalloyed steel is systematically analyzed with a comparison of the differences in the organization and characteristics of microalloyed steel with and without rare earths. The correlation of rare earth elements with the organization and properties of the steel was explored through the organizational analysis of grain size, inclusions, and carbides in the microalloyed steel combined with an analysis of the mechanical properties. The main conclusions are as follows:

1. The addition of rare earths effectively improves the mechanical properties (yield strength, tensile strength, and plasticity) of microalloyed steel.
2. The addition of rare earth elements contributes to the refinement of the organization, the modification of inclusions, and the increase in the carbide volume fraction in microalloyed steel. The combined effect of the multiple factors increases the yield strength of the material.
3. The interaction of rare earth elements with atoms (Nb, C, etc.) in microalloyed steels affects the slip of the dislocations in place, which in turn increases the rate of work hardening of the material and improves its tensile strength.
4. The addition of rare earths increases the volume fraction of carbides in microalloyed steels, as a result of which the pinning effect on dislocations can be increased, increasing the Lüders zone area, which affects the plasticity of the material. In addition, the decrease in the size of inclusions also increases the plasticity of the material.

## Figures and Tables

**Table 1:** Chemical composition of the base steel and RE steel (Fe to balance).

Element (wt.%)	C	Si	S	P	Mn	Al	Nb	Ti	La + Ce	O
Base	0.05	0.17	0.001	0.008	1.55	0.016	0.034	0.012	-	0.0047
RE-added	0.05	0.18	0.001	0.006	1.53	0.023	0.033	0.014	0.0075	0.0011

**Table 2:** The microstructural characteristics of the normalized samples.

Steels	Average of Pearlite Nodule Size (μm)	Average of Ferrite Grain Size (μm)	Pearlite (%)
Base	6.1 ± 0.5	5.0 ± 0.7	21.6 ± 0.8
RE-added	4.3 ± 0.3	4.5 ± 0.8	21.1 ± 0.9

## Data Availability

Data are contained within the article.
